# Interpreting traumatic brain injury severity: analysis of the correlation between Glasgow coma scale and abbreviated injury scale

**DOI:** 10.1007/s00068-025-02909-4

**Published:** 2025-06-27

**Authors:** Puck Domino Monique Niessen, Pieta Krijnen, Henry Alexander Leijdesdorff, Wilco Cornelis Peul, Inger Birgitta Schipper

**Affiliations:** 1 Department of Trauma Surgery, Leiden University Medical Center, Leiden, The Netherlands; 2 Acute Care Network West Netherlands, Leiden, The Netherlands; 3 Department of Trauma Surgery, Haaglanden Medical Center, The Hague, The Netherlands; 4 University Neurosurgical Center Holland, LUMC| HMC| HAGA, Leiden & The Hague, The Netherlands

**Keywords:** Glasgow coma scale, Abbreviated injury scale, Traumatic brain injury, Clinical care, Trauma research

## Abstract

**Purpose:**

Both the Glasgow Coma Scale (GCS) and Abbreviated Injury Scale (AIS) for head injuries quantify traumatic brain injury (TBI) severity. The GCS is based on brain physiology of consciousness, whereas the AIS is an anatomical injury scoring system. This study aimed to describe the correlation of GCS with maximal AIS-Head (maxAIS) and summative AIS-Head (sumAIS) in TBI patients.

**Methods:**

Data of 4996 adult TBI patients admitted to two level 1 trauma centers in the Netherlands between 2015 and 2021 were selected from the regional trauma registry. The association of GCS with maxAIS and sumAIS was quantified using Spearman rank correlation coefficients (r_s_).

**Results:**

For 39% of the patients, the GCS was not documented in the trauma registry. These patients had less severe head injuries than the 3051 patients with documented GCS scores, who were further analyzed. Among those with severe head injuries (AIS-Head ≥ 4), 53% had a GCS score ≥ 13. The GCS showed a weak inverse relationship with both maxAIS and sumAIS (r_s_ -0.33 and − 0.34, respectively, both *p* < 0.001).

**Conclusions:**

The correlation between physiological alterations (GCS) and anatomical brain damage (AIS) in patients with TBI patients, represented by the GCS and AIS respectively, is weak. Additionally, the GCS appears to underestimate the severity of AIS coded severe TBI. Recognizing this limited correlation is important for valid TBI research.

## Introduction

Traumatic brain injury (TBI) is an important global health problem, with considerable socioeconomic impact. TBI has the highest incidence of all common neurological disorders and is a major cause of mortality and morbidity [1, 2]. Assessment of TBI severity is the premier step towards determination of the injury, its accurate treatment and subsequent prognosis. Moreover, precise classification of TBI severity is crucial for valid and reliable TBI research. The severity of the TBI can be assessed using various scoring systems, including the Glasgow Coma Scale and the Abbreviated Injury Scale. The latter is primarily used for registration and research purposes, whereas the Glasgow Coma Scale is mostly applied for (pre) clinical assessment and evaluation, as well as for research. Both scores are registered in the Dutch National Trauma Registry (DNTR), along with other data of all trauma patients who are admitted to Dutch hospitals. These data are used to evaluate national trauma care and to answer research questions.

The Glasgow Coma Scale (GCS), developed in 1974 for the assessment of impaired consciousness and prognostication, is the most widely used classification tool to designate the severity of TBI [3, 4]. The GCS is a dynamic measure of the level of responsiveness, that may vary from moment to moment. Its primary purpose is clinical assessment, and the trend in GCS changes is pivotal for triage decisions. For TBI research purposes, however, the usability of the GCS as an indicator of the extent of neurological damage may be limited, because the score may be influenced by several factors, such as intoxications, medication, timing of assessment, pre-hospital interventions, and concomitant injuries [5, 6].

The Abbreviated Injury Scale (AIS) is an anatomic injury coding system that has been in use since 1990. The last digit of each diagnosis code (AIS score) represents the injury severity, ranging from 1 (minor) to 6 (fatal) [7, 8]. The AIS Head score is often used additional to the GCS to select patients for clinical trials or when the GCS score is missing [9]. Contrary to the GCS, the AIS score is determined after a complete diagnostic workup through a review of the patient’s records including neuroradiology and intraoperative findings. As the AIS-Head score does not depend on time of assessment or other time-related factors, it may be an alternative scoring tool for determination of the severity of brain injury in TBI research. The maximum AIS (maxAIS) is the most used variable. However, the summative AIS (sumAIS), encompassing not only the most severe head injury of the patient but adding up the severity of all head injuries, may potentially serve as a superior variable.

This study aimed to investigate the association of the GCS with both maxAIS-Head and sumAIS-Head in TBI patients and various subgroups.

## Methods

### Trauma registry

The DNTR was established in 2007 and is used to organize, evaluate, and improve trauma care. All patients treated in the Emergency Department (ED) within 48 hours after trauma and admitted to the hospital, or deceased in the ED are registered regionally in the DNTR [10, 11]. This inclusive registry is now one of the most extensive trauma registries worldwide and a major data source for assessment of trauma care quality and trauma research. For this study, DNTR data of a cohort of TBI patients from the Trauma Region West Netherlands (TRWN) was retrospectively analyzed. No approval from the Institutional Review Boards was needed for the use of the anonymized registry data.

### Study population

The study included adult TBI patients who were admitted between 2015 and 2021 to one of two level 1 trauma hospitals in TRWN: the Leiden University Medical Center (LUMC) and Haaglanden Medical Center (HMC). TBI included intracranial vessel injuries (AIS codes 120199.3–122704.3), cranial nerve injuries (AIS codes 130202.2–132699.2), internal organ (brain stem, cerebellum, cerebrum) injuries (AIS codes 140202.5–140799.3), skull fractures (AIS codes 150000.2–150408.4), and concussive injuries (AIS codes 161000.1–161013.5) [5, 12]. Patients without any registered data in the ED and patients with non-specified TBI severity (AIS Head = 9) were excluded from the analysis.

### Study parameters

#### GCS

The GCS score ranges from 3 to 15, categorizing scores between 13 and 15 as mild brain injuries, scores between 9 and 12 as moderate injuries, and scores between 3 and 8 as severe injuries. GCS scores documented at the ED were analyzed. The GCS scores in the ED are determined by various medical professionals, including emergency physicians, trauma surgeons and neurosurgeons. If one or more components of the GCS score (Eye, Motor and/or Verbal response) were not recorded at the ED, the GCS score could not be calculated and was registered as missing in the DNTR. For patients who were already intubated and/or sedated upon arrival at the Emergency Department (ED), the initial values for Eye (E), Motor (M), and Verbal (V) responses recorded at the ED were retrieved and taken from the patient’s medical records. Typically, these values in the medical records were E = 1 (no eye-opening), M = 1 (no motor response), and V = 1 (no verbal response), but not for all cases.

#### AIS

Two AIS variables were constructed. First, the maximum AIS (maxAIS), is defined as the highest AIS Head score of all head injuries of a patient [8, 13]. This maxAIS was categorized as severe (4–6); moderate (3); or mild (1–2). Second, the summative AIS (sumAIS) was defined as the sum of the AIS Head scores of all head injuries of a patient.

#### Other study parameters

Furthermore, the number of AIS coded head injuries was determined. AIS scores of injuries in other body regions were used to define whether the patient had isolated head injury or multiple injuries (AIS 1–5), and specifically the patients with serious concomitant injury (AIS ≥ 3). The Injury Severity Score (ISS) was used to express the overall injury severity [8]. Other study parameters included sex, age, and injury characteristics. Age was categorized into young adults (18–35), middle-aged adults (36–50), pre-elderly (51–65) and elderly (> 65 years). The outcome of care was in-hospital mortality.

### Statistical analysis

Patient and injury characteristics were described using summary statistics. The characteristics were compared between the patients with documented and missing GCS using an independent Samples T-Test for normally distributed continuous data and a Chi-Square Test for categorical data. The patients with missing GCS were excluded from further analyses. The association of the GCS with maxAIS and sumAIS Head scores was graphically presented and quantified using Spearman rank correlation coefficients, for the total group and subgroups of patients. All analyses were performed using IBM SPSS Statistics for Windows, version 25.0 (IBM Corp., Armonk, N.Y., USA). P-values < 0.05 were considered statistically significant.

## Results

### Demographic and clinical characteristics

A total of 5302 adult patients with TBI were admitted to the participating centers between 2015 and 2021. For 305 patients no ED data were available in the DNTR and TBI severity was not specified for one patient. Thus, 4996 patients were analyzed, including 2048 women and 2948 men, with an average age of 60.0 years (Table 1).

**Table 1:** Patient and injury characteristics of 4996 patients with and without documented GCS

*Patient characteristics*	All Patients (n = 4996)	Patients with documented GCS (n = 3051)	Patients with missing GCS (n = 1945)	P value
Demographics				
Sex, n (%)				0.020
Female	2048 (41.0)	1290 (42.3)	758 (39.0)	
Male	2948 (59.0)	1761 (57.7)	1187 (61.0)	
Age in years, mean (SD)	60.0 [21.2]	60.6 [20.8]	59.1 [21.7]	0.016
Age, years, n (%)				0.013
18–35	863 (17.3)	499 (16.3)	364 (18.7)	
36–50	743 (14.9)	429 (14.1)	314 (16.1)	
51–65	1090 (21.8)	682 (22.3)	408 (21.0)	
> 65	2300 (46.0)	1441 (47.2)	859 (44.2)	
Injury characteristics				
GCS, n (%)				
≤ 8		268 (8.8)		
9–12		229 (7.5)		
13–14		687 (22.5)		
15		1867 (61.2)		
Summative AIS (sumAIS) Head, n (%)				< 0.001
1–5	3780 (75.7)	2207 (72.3)	1573 (80.9)	
6–10	701 (14.0)	495 (16.2)	206 (10.6)	
11–15	362 (7.2)	248 (8.1)	114 (5.9)	
16–19	83 (1.7)	56 (1.8)	27 (1.4)	
20 +	70 (1.4)	45 (1.5)	25 (1.3)	
Maximum AIS (maxAIS) Head, n (%)				< 0.001
1	1952 (39.1)	1023 (33.5)	929 (47.8)	
2	1127 (22.6)	786 (25.8)	341 (17.5)	
3	1136 (22.7)	742 (24.3)	394 (20.3)	
4	417 (8.3)	255 (8.4)	162 (8.3)	
5	364 (7.3)	245 (8.0)	119 (6.1)	
Type of head trauma, n (%)				0.045
Blunt injury	4931 (99.4)	3014 (99.2)	1917 (99.7)	
Penetrating injury	29 (0.6)	23 (0.8)	6 (0.3)	
No. of AIS Head injury codes, n (%)				< 0.001
1	3125 (62.5)	1765 (57.8)	1360 (69.9)	
2–5	1732 (34.7)	1184 (38.8)	548 (28.2)	
> 5	139 (2.8)	102 (3.3)	37 (1.9)	
Isolated head injury, n (%)	1253 (25.1)	703 (23.0)	550 (28.3)	< 0.001
Serious concomitant injury (AIS ≥ 3), n (%)	935 (18.7)	616 (20.2)	319 (16.4)	0.001
ISS, n (%)				< 0.001
< 16	3701 (74.1)	2195 (71.9)	1506 (77.4)	
≥ 16	1295 (25.9)	856 (28.1)	439 (22.6)	
Mechanism of TBI, n (%)				< 0.001
Inflicted by others/violence	221 (4.5)	102 (3.4)	119 (6.2)	
Traffic accident	1718 (34.6)	1113 (36.6)	605 (31.5)	
Occupational accident	92 (1.8)	64 (2.1)	28 (1.5)	
Private accident	2656 (53.2)	1595 (52.5)	1061 (55.2)	
Sport	120 (2.4)	88 (2.9)	32 (1.7)	
Self-mutilation/TS	45 (0.9)	27 (0.9)	18 (0.9)	
Other mechanisms	108 (2.2)	48 (1.6)	60 (3.1)	
Diagnosis of TBI^1^, n (%)				
Concussive injury	3470 (69.4)	2114 (69.3)	1356 (69.7)	0.749
Internal organ injury	2167 (43.4)	819 (26.8)	732 (37.6)	< 0.001
Skeletal injury	1198 (24.0)	1435 (47.0)	379 (19.5)	< 0.001
Intracranial vessel injury	29 (0.6)	20 (0.7)	9 (0.5)	0.382
Cranial nerve injury	53 (1.1)	40 (1.3)	13 (0.7)	0.031
Outcomes				
In-hospital mortality, n (%)	274 (5.5)	187 (6.1)	86 (4.4)	0.010

*GCS*, Glasgow Coma Scale; *AIS*, Abbreviated Injury Score; *ISS*, Injury Severity Score; *TBI*, Traumatic Brain Injury; *TS*, tentamen suicidii

^1^Patients can have multiple head diagnoses (coded by AIS), so the summed percentages exceed 100%

In 1952 (39.1%) patients the maxAIS Head was 1, and 781 patients (15.6%) had severe head injury classified as maxAIS ≥ 4. The GCS score at the ED was recorded for 3501 (61.1%) patients. A GCS score of 15 was found in 1867 (61.2%) of these patients, and 268 (8.8%) of these patients had a GCS score ≤ 8. The most frequently diagnosed TBIs were concussive injuries (69.4%) and internal organ injuries (43.4%) of the brain. Most (62.5%) of the 4996 patients had a single head injury. All but 29 (0.6%) TBI patients had sustained a blunt trauma mechanism. 25% of the patients presented with isolated head injuries and 18.7% with serious injury (AIS ≥ 3) in other body regions than the head.

### Missing GCS in relation to patient and injury characteristics and outcomes of care

Patients with missing GCS scores differed from those with documented GCS concerning patient and injury characteristics (Table 1). In particular, patients with missing GCS had a lower number of head injury diagnoses and less severe head injuries according to the AIS. Consequently, they had a lower in-hospital mortality rate. These patients were excluded from further analysis.

### Association between GCS and AIS

**Fig. 1:**
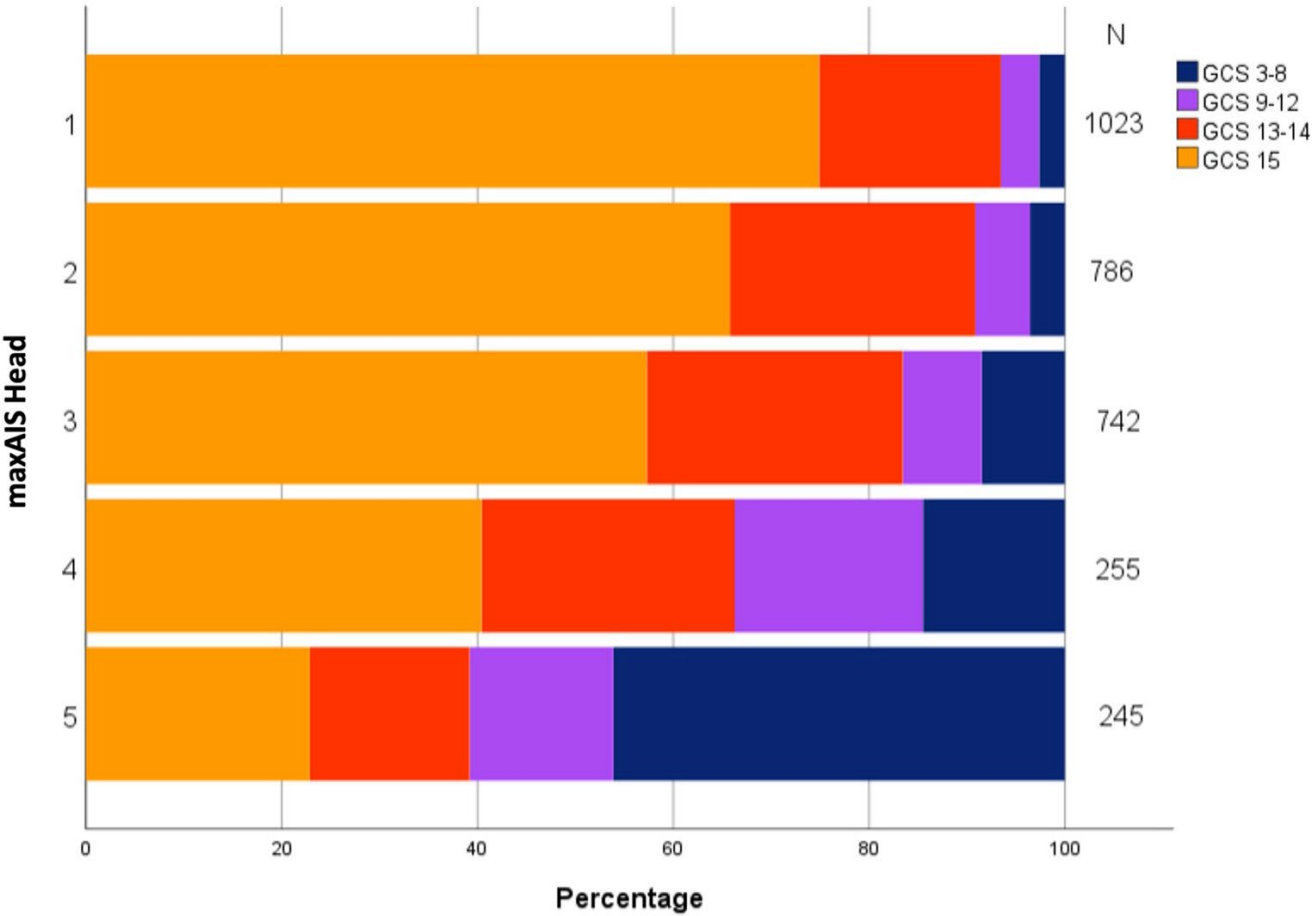
Distribution of GCS category by maxAIS Head category

**Fig. 2:**
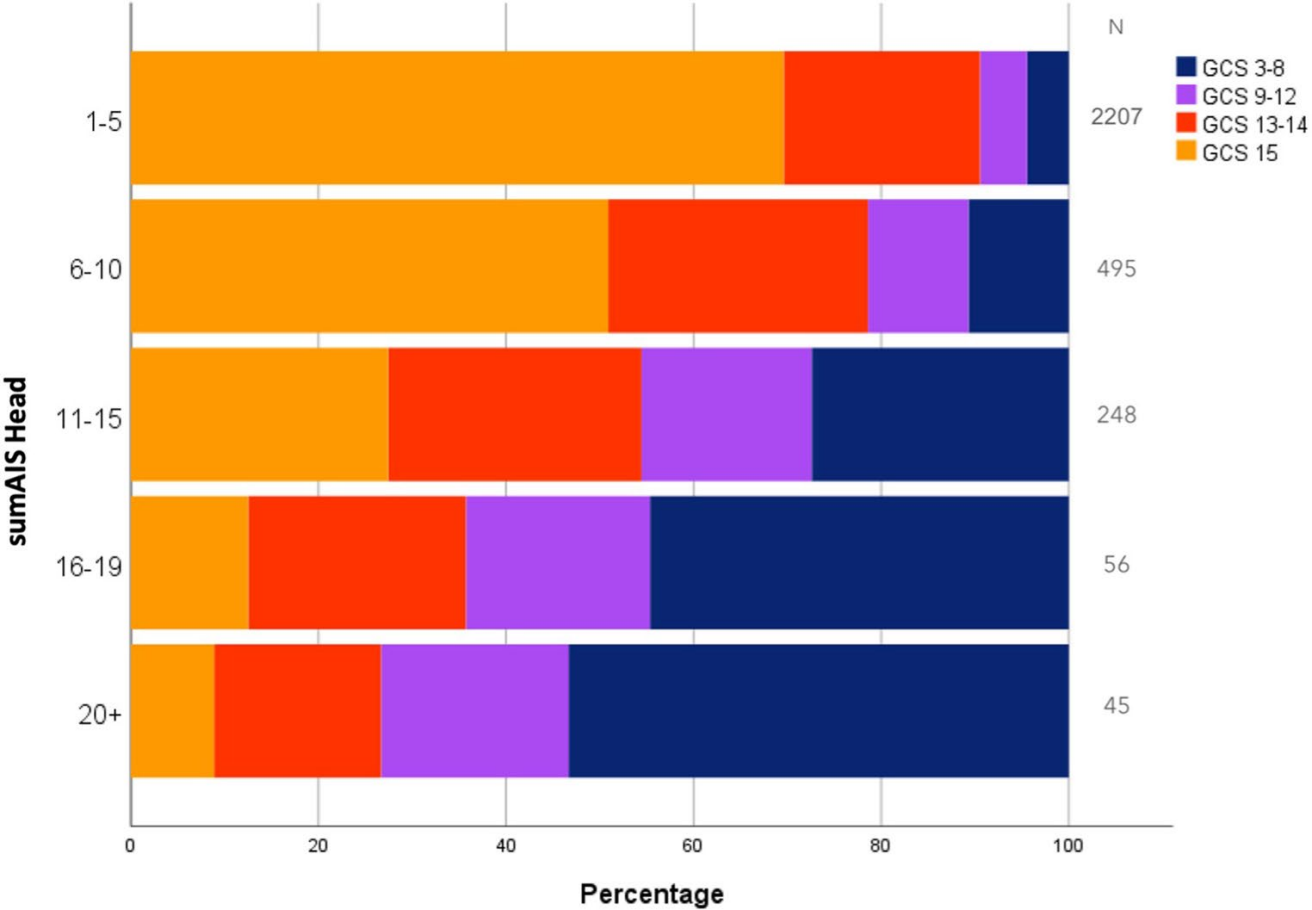
Distribution of GCS category by sumAIS Head category

Most (67.0%) patients with maxAIS-Head scores 1–3 had a GCS score of 15, and 22.8% had GCS scores of 13 or 14 (Fig. 1). Of the patients with a maxAIS-Head of 4, 40.4% had a GCS of 15 and 25.9% a GCS of 13–14. In the patients with a maxAIS Head of 5, these percentages were respectively 22.9% and 16.3%. In Fig. 2, the distribution of GCS by sumAIS-Head is displayed, where 10.9% of the patients with sumAIS Head > 15 had a GCS score of 15, and 20.8% had a GCS score of 13 or 14.

The GCS had a weak inverse relationship with both maxAIS Head and sumAIS Head in the total patient group (Spearman rank correlation coefficient − 0.33 and − 0.34, respectively, both *p* < 0.001; Tables 2 and 3). Since the maxAIS Head and sumAIS Head are similar in patients with a single AIS Head code, we also estimated the correlation between GCS and AIS head scores in the subgroup of 1286 patients with multiple head injuries. For these patients, the correlation between GCS and maxAIS Head was − 0.41 (*p* < 0.001) and between GCS and sumAIS Head − 0.44 (*p* < 0.001).

**Table 2:** Spearman rank correlation coefficients (r_s_) between GCS and **MaxAIS** head in all patients and subgroups

Group	*n*	*r*_s_	*p*-value
All patients	3051	−0.334	< 0,001
Age			
18–35 yrs	499	−0.342	< 0.001
36–50 yrs	429	−0.244	< 0.001
51–65 yrs	682	−0.326	< 0.001
65 + yrs	1441	−0.372	< 0.001
Sex			
Female	290	−0.335	< 0.001
Male	1761	−0.333	< 0.001
Diagnosis			
Concussive injury	2114	−0.221	< 0.001
Internal organ injury	1435	−0.374	< 0.001
Skeletal injury	819	−0.470	< 0.001
Intracranial vessel injury	20	−0.436	0.055
Cranial nerve injury	40	−0.531	0.001
Type of TBI			
Blunt injury	3014	−0.331	< 0.001
Penetrating injury	23	−0.575	0.004
Extensiveness of injury			
Isolated head injury	703	−0.267	< 0.001
No isolated head injury	2348	−0.359	< 0.001
Concomitant injury (AIS ≥ 3)	616	−0.480	< 0.001

*GCS* Glasgow Coma Scale, *AIS* Abbreviated Injury Score, *maxAIS Head* maximum AIS of the head, *TBI* Traumatic Brain Injury

In other subgroup analyses, the correlation coefficients between GCS and maxAIS or sumAIS Head scores were highest for patients with penetrating injury (−0.58, *p* = 0.004 and − 0.61, *p* = 0.002, respectively) and lowest for patients with concussive injury (−0.22 and − 0.23 respectively, both *p* < 0.001; Tables 2 and 3). Regarding the diagnosis subgroups, cranial nerve injury showed the strongest correlation between GCS and maxAIS Head (−0.53, *p* = 0.001), and between GCS and sumAIS Head (−0.57, *p* < 0.001). Patients with multiple injuries (−0.36, *p* < 0.001) and specifically patients with serious concomitant injury (−0.48, *p* < 0.001) presented a stronger correlation between GCS and maxAIS Head than patients with isolated head injury (−0.27, *p* < 0.001). This was also seen for the correlation between GCS and sumAIS Head. Regarding age groups, patients aged 36 to 50 years showed the weakest correlations. The correlations for males and females were similar.

**Table 3:** Spearman rank correlation coefficients (r_s_) between GCS and **SumAIS** head in all patients and subgroups

Group	*n*	*r*_s_	*p*-value
All patients	3051	−0.344	< 0,001
Age			
18–35 yrs	499	−0.318	< 0.001
36–50 yrs	429	−0.250	< 0.001
51–65 yrs	682	−0.319	< 0.001
65 + yrs	1441	−0.403	< 0.001
Sex			
Female	1290	−0.347	< 0.001
Male	1761	−0.341	< 0.001
Diagnosis			
Concussive injury	2114	−0.226	< 0.001
Internal organ injury	1435	−0.410	< 0.001
Skeletal injury	819	−0.462	< 0.001
Intracranial vessel injury	20	−0.286	0.145
Cranial nerve injury	40	−0.567	< 0.001
Type of TBI			
Blunt injury	3014	−0.341	< 0.001
Penetrating injury	23	−0.612	0.002
Extensiveness of injury			
Isolated head injury	703	−0.265	< 0.001
No isolated head injury	2348	−0.366	< 0.001
Concomitant injury (AIS ≥ 3)	616	−0.475	< 0.001

*GCS* Glasgow Coma Scale, *AIS* Abbreviated Injury Score, *sumAIS Head* summative AIS of the head, *TBI* Traumatic Brain Injury

## Discussion

The present study aimed to describe the correlation of GCS with maximal AIS Head and summative AIS Head, in TBI patients and subgroups. The results demonstrated that the GCS documented at the ED hardly correlates with both studied AIS-Head parameters. The Spearman rank correlation coefficients between GCS and maxAIS/sumAIS-Head in the total TBI patient group and all subgroups varied between 0.2 and 0.6, indicating that there was at best a moderate correlation between the two injury scales.

Similar to our findings, Demetriades et al. showed a Stuart’s Tau-C correlation coefficient of −0.35 and Rogers et al. a Pearson correlation coefficient of −0.40 [14, 15]. Hudak et al. even found a non-significant Spearman correlation coefficient of −0.04, indicating no correlation between GCS and AIS Head [16]. This limited association between the GCS and AIS is likely to be attributed to the respective methods used for assessing injury severity and the physiological focus. The GCS evaluates injury severity based on clinical neurological signs of (un)consciousness at a specific moment, whereas the AIS is determined at discharge following a comprehensive evaluation and considers the severity of injury specifically to the anatomical regions of the head. Moreover, the difference in timing of the measurement of GCS and AIS could contribute to the limited correlation, as the GCS is assessed upon admission, or even in sedated/intubated patients, while the AIS is determined after a comprehensive work-up, including imaging. Some traumatic brain injuries, such as intracranial hemorrhages and diffuse axonal injury, may initially present with normal consciousness but can deteriorate over time.

Patients with multiple injuries and specifically polytrauma patients with serious concomitant injury presented a stronger correlation between GCS and AIS Head than patients with isolated head injury. A possible explanation for this stronger correlation is that the group with multiple injuries is likely to include a higher proportion of severely injured patients, and in these patients, a more homogeneous clinical presentation may emerge, characterized by both low GCS and high AIS Head scores. Additionally, since our study indicates that GCS may underestimate the severity of anatomical head injury. It is conceivable that when the GCS deteriorates due to additional injuries the correlation with AIS may improve.

The strongest correlation between GCS and AIS Head was found in the small subgroup of patients with penetrating TBI. This outcome contrasts with our initial assumptions; on the one hand does moderate penetrating injury often results in a more focal injury with anatomical functional damage, potentially not directly impacting the GCS, but resulting in a high AIS due to the damaged structure. Alternatively, blunt injury can in severe cases cause diffuse axonal injury (DAI) with brainstem damage (high AIS) and direct loss of consciousness. However, the limited subgroup size of 23 patients with TBI resulting from penetrating trauma is insufficient to draw definitive conclusions on this observed correlation.

The results of the current study show a remarkable discrepancy between AIS-Head and GCS. Of the patients with a maxAIS score of 4 or 5, 53% had a GCS score ≥ 13 and 70% had a GCS score > 8. Thus, mildly decreased GCS scores seem to potentially underestimate the injury severity in many patients with serious head injuries. This result was found previously by Heather et al. in children aged < 5 years, and by Mica et al. in multiple-injured patients (ISS ≥ 16) [17, 18]. Clinicians should be aware of this possible underestimation when using the GCS at ED admittance. Alternatively, the AIS coding may overvalue the severity of anatomical findings.

Severe TBI is internationally classified as a GCS score between 3 and 8. Clinicians and scientists during the recent InTBIr 2024 meeting in Washington questioned whether patients with moderate TBI, according to the GCS, may actually often relate to more severe (anatomical) brain injury, which in turn may predict a worse outcome than expected from mild or moderate TBI. After all, the GCS is a dynamic scale for consciousness with restricted insights into the anatomical damage and the severity of the brain injury. Therefore, the AIS-Head, or an equivalent, should be considered as an additional clinical classification tool to the GCS.

In nearly 40% of the patients, the GCS value was not documented at the ED. The missing GCS values seemed to be associated with less extensive and less severe TBI according to the AIS and, therefore, to more a favorable outcome in terms of in-hospital mortality. A possible explanation for these observations is that more stable patients may be perceived as less at risk for clinical deterioration, and thus their clinical status might be less meticulously documented. However, our results show that one may not assume that a patient’s consciousness was not impaired if the GCS was not documented. Extensive measurements and documentation might also be missing for major trauma patients, because of the urgent setting. This assumption is consistent with the findings of O’Reilly et al., who– in contrast to our findings– found that patients with missing GCS scores had more severe TBI and that missing GCS scores correlated with higher in-hospital mortality [19].

To our knowledge, this is the first study assessing the association between the GCS and the sumAIS Head. We hypothesized that the sumAIS might be a more accurate measure for assessing head injury severity than the maxAIS since it encompasses all head injuries sustained and not only the one with the highest AIS score. However, we observed comparable correlation results for sumAIS and maxAIS in all patients. Since this may be explained by the fact that most patients (57.8%) only had a single AIS code and therefore identical maxAIS and sumAIS scores, we also analyzed the correlation in the subgroup of patients with multiple head AIS codes. In these patients the correlations between GCS and maxAIS, and GCS and sumAIS were also similar: respectively − 0.41 and − 0.44. The strong correlation between sumAIS and maxAIS (r_s_ = 0.94) is an interesting finding by itself. More research on this matter is necessary to ascertain if sumAIS potentially enables more precise estimation of traumatic brain injury severity.

### Limitations

All limitations associated with retrospective research apply to this study. The fact that the GCS was missing for 38.9% of the patients has introduced a significant selection bias. Nevertheless, excluding these patients from the analyses was a deliberate choice. We considered supplementing missing GCS data in the ED with available pre-hospital GCS data but decided against it, to avoid bias induced by differences in timing of the GCS classification. Furthermore, we considered imputing missing GCS scores with the maximum GCS score. In that way, we would assume a more favorable neurological course on average for patients with missing GCS scores, which was deemed unjustified. Consequently, using only the patients with available GCS scores appeared the most valid approach. The fact that GCS data are missing selectively and do so in many cases in trauma registries, underlines the importance of additionally using AIS to determine injury severity in TBI research.

The GCS scores documented in the DNTR are assessed by a diverse range of medical professionals with varying levels of experience, which may have introduced variability. Previous studies indicate high interobserver agreement for GCS scores determined by experienced medical staff; however, less agreement has been shown evident when the GCS score was determined by less experienced practitioners [20–22]. Substantial interobserver variability was also found in the assessment of agreement on AIS scores [23]. The AIS scores used in our study were documented in the DNTR by trained trauma registrars, which may have helped considerably to reduce the variability.

## Conclusion

The current study indicates that the correlation between the GCS and the maxAIS or sumAIS-Head in patients with TBI is limited. Additionally, the GCS appears to underestimate the severity of major anatomical TBI. Therefore, in the future, the AIS-Head may be considered as an additional clinical classification tool. Given that accurate and consistent assessment of head injuries is crucial for interpretation of anatomy and physiology in clinical practice and valid TBI research, it is important to recognize the strengths and limitations of both classifications for use in trauma care. Therefore, GCS and AIS should not be considered interchangeable in TBI research studies. Currently, the acute GCS scores seem often undocumented in the medical files of TBI patients. A future prognostic study exploring the synergistic use of the GCS as a measure of consciousness and AIS as an anatomical scale could enhance our understanding of brain damage severity and improve the predictive value for functional outcomes. As discussed during the recent InTBIr meeting in Washington (2024) the currently used GCS and Pupillary response alone in neurotraumatology does not have any prognostic value and the classification in mild, moderate and severe TBI underestimates the physical, cognitive and social consequences for patients. Several working groups will discuss an alternative TBI classification, combining the classical GCS score with Biomarkers and Anatomical Pathology confirmed by radiological parameters.

## Data Availability

Data from this study are available from the RTR, but not available in a public archive. The analytic code used to conduct the analyses presented in this study are not available in a public repository, but it may be obtained by emailing Prof. Dr. Schipper (I.B.Schipper@lumc.nl).
